# THE MEDIATING ROLE OF PERCEIVED MASTERY ON THE RELATIONSHIP BETWEEN SELF-PERCEPTIONS OF AGING AND COGNITION

**DOI:** 10.1093/geroni/igae098.3280

**Published:** 2024-12-31

**Authors:** Hannah Apostolou, Michael Buxton, Valerie Luskey, M Lindsey Jacobs

**Affiliations:** University of Alabama, Northport, Alabama, United States; University of Alabama, Tuscaloosa, Alabama, United States; University of Alabama, Tuscaloosa, Alabama, United States; The University of Alabama, Tuscaloosa, Alabama, United States

## Abstract

Internalized ageism can be understood as a negative perception of one’s own aging experience. Negative perceptions of aging may lead to disengagement in daily tasks and exacerbate decreases in cognition. Positive self-perceptions of aging, however, may serve as a protective mechanism for cognition. Additionally, the relationship between self-perceptions of aging and cognition may be impacted by one’s sense of control in areas such as perceived mastery. Researchers used data from the 2020 Health and Retirement Study to examine the relationship between self-perceptions of aging on total cognition and the mediating influence of perceived mastery in a sample of older Americans (n=4369). Specific measures from the Psychosocial and Lifestyle Questionnaire and cognitive battery were selected to conduct the analyses in PROCESS for SPSS Version 4.2. The results showed that there was a significant total effect between self-perceptions of aging and total cognition (B=0.018, p< 0.001), and path a (i.e., self-perceptions of aging on perceived mastery) (B=0.004, p< 0.001) and path b (i.e., perceived mastery on total cognition) (B=0.795, p>0.001) were both significant. The direct effect (B=0.014, p< 0.001) was significant when adding perceived mastery to the relationship between self-perceptions of aging and total cognition. This suggests that more positively rated self-perceptions of aging are associated with higher total cognition scores independently, and this relationship may also operate via perceived mastery. Interventions aimed at increasing positive attitudes towards one’s own aging experience could lead to increased cognitive functioning. Further, skills training aimed at increasing one’s sense of control may further benefit cognitive outcomes.

